# The oral mucosa in leprosy: a clinical and histopathological study

**DOI:** 10.1016/S1808-8694(15)30962-9

**Published:** 2015-10-19

**Authors:** Marilda Aparecida Milanez Morgado de Abreu, Nilceo Schwery Michalany, Luc Louis Maurice Weckx, Dalva Regina Neto Pimentel, Cleonice Hitomi Watashi Hirata, Maurício Mota de Avelar Alchorne

**Affiliations:** a MS. Post-Graduate student - UNIFESP/EPM; b MS. Assistant Professor – Department of Pathology - UNIFESP/EPM; c Associate Professor, Full Professor of the Department of Otorhinolaryngology and Human Communications Disorders and Head of the Department of Otorhinolaryngology and Head and Neck Surgery - UNIFESP/EPM; d MS. Post-graduate student - UNIFESP/EPM; e MS. PhD, Head of the Department of Stomatolgy - UNIFESP/EPM; f Associate Professor, Full Professor – Department of Dermatology - UNIFESP/EPM. Federal University of São Paulo – Paulista School of Medicine (UNIFESP/EPM)

**Keywords:** leprosy, histopathology, oral mucosa

## Abstract

**Introduction:**

Multibacillary leprosy may involve the oral mucosa, with or without apparent lesions. There are few studies that deal with this issue in the era of multidrug therapy.

**Aim:**

To assess the frequence of oral mucosa involvement in multibacillary leprosy patients.

**Patients and Methods:**

A transversal study with twenty non-treated multibacillary leprosy patients. The patients were treated in Dracena, São Paulo, between 2000 and 2002. Clinical examination of the oral mucosa was carried out. All patients were submitted to jugal mucosa, soft palate and tongue biopsies, in altered or in pre-established sites. The cross-sections were stained by techniques of hematoxilin-eosin and Ziehl-Neelsen. Granuloma and alcohol-acid-resistant bacilli findings determined the specific histopathological involvement.

**Results:**

The study involved 19 patients with an average of 2.5 years of disease progression. Specific histopathological involvement occurred in the tongue and soft palate of one lepromatous patient with an apparently normal oral mucosa.

**Conclusions:**

(1) Clinical alterations in the oral mucosa does not imply disease involvement, it is necessary to have histopathological confirmation. (2) Apparent specific clinical alterations are rare. (3) The clinically normal oral mucosa can show specific histopathological involvement.

## INTRODUCTION

Leprosy is a chronic infectious contagious disease caused by Mycobacterium leprae (M. leprae). It affects mainly the skin and peripheral nerves and also internal organs and mucosa. The initial form of the disease is the undetermined form, which may resolve spontaneously or progress to a wide spectrum of clinical presentations. These reflect different immune cellular responses to M. leprae, pre-determined by the innate capability of the host to resist infection. Thus, the disease may remain limited, in the tuberculoid pole (TT), upgrade to disseminated forms - the virchowian pole (VV) - or assume an intermediate position between these two poles, the so-called dimorphous group. Depending of its proximity to one or the other pole, the dimorphous group may be subdivided into the dimorphous-tuberculoid form (DT), the dimorphous-dimorphous (DD) form or the dimorphous-virchowian form (DV)1. Operationally and therapeutically, in 19822 the World Health Organization, classified as patients with a positive Mitsuda test (immunity against the bacillus) and a bacilloscopy below 2 as paucibacillar, and patients with a negative Mitsuda test (no immunity against the bacillus) and bacilloscopy over 2 as multibacillar. Clinical forms I, TT and DT are paucibacillar clinical forms DD, DV and VV are multibacillar^2^.

In contrast to cutaneous manifestations that are well described in medical literature, there are few published studies dealing with the oral manifestations of leprosy and a lack of detailed descriptions in standard textbooks. The majority of the references are outdated, of a time when patient's disease progressed for lack of efficient treatments for the disease. This subject gained interest due to the fact that the upper airways are the main entry door for the bacillus and the route for bacillary elimination. The nasal mucosa is affected in initial stages of the disease, usually preceding cutaneous manifestations^3^. The oral cavity may be contaminated by bacilli present in rhinopharyngeal secretions, however, notwithstanding this contamination, oral cavity is resistant to lesions. These are almost only restricted to multibacillar patients in advanced stages of disease^4^, ^5^, ^6^, ^7^, ^8^, ^9^, ^10^, ^11^, which suggests that bacillary invasion of the oral cavity results from bacillemia from bacterial dissemination and multiplication^12^, ^13^. However, the oral cavity with no evident injuries may be affected in less advanced stages of the disease. Bacilloscopic examinations of the clinically normal mucosa done by Hubscher et al. in 1979^14^ detected bacilli in 7 of 17 specimens of the tongue, the hard palate and the gingiva. Studies by Brazil et al. in 1973^15^ detected bacilli in 16 of 112 specimens of the soft palate, of which 4 biopsied cases had granulomas. Kumar et al., in 1988^16^, in histopathological exams of the clinically normal mucosa, found granulomas in 11 specimens and bacilli in 4 specimens of the cheek in 17 biopsied cases. Granulomas were also observed in the hard palate in 9 specimens, and bacilli in 4 specimens of 14 biopsied cases. Sharma et al. in 1993^12^, histopathologically detected a perivascular lymphomononuclear infiltrate with bacilli in 1 case and bacilli with no inflammatory reaction in 2 cases of 5 specimens of clinically normal tongues. In recent literature, in the era of multiple drug therapy, there is still insufficient data on oral cavity involvement in leprosy. A current study on this theme is of extreme interest for stomatology, as this disease is still a major public health problem in our country^17^. Therefore, we proposed a clinical and histopathological study of untreated leprosy patients to verify the frequency of oral cavity involvement in this disease

## PATIENTS AND METHODS

A cross-sectional study of 20 patients with leprosy seen consecutively in the city of Dracena, located in western Sao Paulo state, between 2000 and 2002. The study included multibacillar patients, regardless of gender, color, age or duration of the disease. Previous treatment was an exclusion criterion. The study only started after design analysis and approval by the Sao Paulo Federal University Research Ethics Committee, protocol number 498/01, with prior participant agreement upon signing a free and informed consent form. Diagnosis of leprosy was based on clinical, bacilloscopic and histopathological criteria according to Ridley & Jopling's classification (1962)^1^ of clinical forms. The patients were generally grouped as multibacillar according to World Health Organization 1982^2^ criteria. The oral cavity was carefully examined, following a systematic and ordered methodology including inspection and palpation to identify clinical findings; if there were no findings, the patient was labeled clinically normal. Biopsies were made in all patients in three areas of the oral cavity both in clinically normal and in altered mucosa, a total of 60 specimens. The biopsy points were: the mid-point of the cheek, 1 cm from the tip of the tongue, and the soft palate close to the base of the uvula. Biopsies were done under local spray or infiltration lidocaine 2% anesthesia using a digestive endoscopic 1.8 mm clamp and a 3 mm punch (in the soft palate on the digestive endoscopic clamp was used) and hemostasis was obtained by compression or suture. Mucosal fragments were fixed in formaldehyde 10%, included in paraffin and sections were hematoxilineosine (HE) and Ziehl-Neelsen (ZN) stained. Finally, one pathologist only assessed the specimens under common optical microscopy at 40 times and 1,000 times magnification. The evaluation of results was based on a clinical and histopathological interpretation. Specific leprosy involvement of the oral cavity was defined as the presence of acid-alcohol resistant bacilli in the ZN stained sections, and granulomas in HE stained sections, regardless of the presence or not of visible lesions.

## RESULTS

There were 13 male and 7 female patients, mostly caucasian aged between 27 and 74 years, average 53.6 years. Disease progression time varied between 3 months to 14 years, average 2.5 years; 12 patients had presented symptoms in the last 3 months to 1 year, 6 presented symptoms in the last 2 to 4 years and only 2 presented symptoms in the last 10 to 14 years. Clinical forms were 11 VV, 7 DV and 2 DD. Clinical and histopathological examination results of the oral cavity are shown in Table 1.

**Table 1:** Clinical examination of the oral cavity according to the clinical form. Dracena, 2002.

Clinical form	Cheek		Tongue		Soft palate	
	Alteration (type)	Normal	Alteration	Normal	Alteration	Normal
DD	1 (erythematous macula)	1	2 (fissure & edema)		1 (papule)	1
DV	1 (hypochromic macula)	6	1 (fissured & geographic)	6		7
VV	1 (hypochromic macula)	9	1 (fissured & geographic) 3 (fissures)	5		11
	1 (erythematous macula)		1 (fissures, infiltration & atrophy) 1 (fissures & papula)			
Total	4	16	9	11	1	19

## DISCUSSION

The absence of granulomas and acid-alcohol resistant bacilli in the histopathology of the clinically detected lesions of the oral cavity demonstrates the non-specific nature of these lesions. This is in accordance with Port (1965)^6^ and Brazil et al. (1974)^8^ when they state that no lesion in the oral cavity is pathognomonic of leprosy. These lesions should be biopsied and analyzed histopathologically; the association of acid-alcohol resistant bacilli and a granulomatous inflammatory reaction are the only criteria that allow us a diagnosis of leprosy. Thus many non-specific lesions may have been associated with leprosy in the past, as the original studies of the diagnosis of such lesions was established only through the clinical examination^4^, ^5^, ^6^, ^7^ or bacilloscopy^8^, ^9^. Few authors did histopathological examination of detected lesions^10^, ^12^, ^13^, ^16^. This may be one of the explanations for the disagreement in frequency rates of oral involvement in leprosy seen in literature, which varies from absent up to 57.5%^4^, ^5^, ^6^, ^7^, ^8^, ^9^, ^10^, ^11^. The decision to biopsy three areas was to increase the positive result rate. The choice of biopsy areas was based on the proven possibility of involvement in those areas, regardless of the presence or absence of visible lesions^12^, ^14^, ^15^, ^16^. The clinically normal mucosa of the soft palate, in particular, had not yet been studied using histopathology; although Brazil et al. in 1973^15^ had demonstrated high rates of positive bacilloscopies in this site, these authors had done histopathology in only 4 cases, guided by positive bacilloscopies. The importance of studying this site is based on many past studies that have established the soft palate as being the most frequently involved oral site in this disease^5^, ^8^, ^15^, ^18^, ^19^. It was shown that the oral cavity may be involved even in the absence of visible lesions, which is in agreement with literature^12^, ^14^, ^15^, ^16^. And this may occur in less advanced stages of the disease, as was the case of a patient in this study, which had presented symptoms of the disease only in the past 3 years. This agrees with Brasil et al. (1974)^8^, who stated that oral cavity involvement is not typical of long standing cases. In these cases oral cavity involvement remains clinically hidden, and may only be seen histopathologically. It is, therefore, evident that if such involvement exists, and the disease is not diagnosed, progressing with no effective treatment, a specific visible lesion will eventually appear. Today, improved control of leprosy since the introduction of multiple drug treatment, has dramatically reduced the frequency of oral lesions in this disease. The true meaning of the presence of M. leprae in the absence of oral cavity lesions is still not understood. Tentative explanations generate many question: higher temperatures, accelerated epithelial renewal, local action of salivary enzymes, local immunological factors… Whatever the importance of this fact, it seems unquestionable from the epidemiological standpoint, as bacilli can be eliminated into environment by talking, spitting, sneezing or coughing. It should also be emphasized that medical personnel should protect themselves during invasive procedures in the mouth of individuals with multibacillary leprosy. Another question is why M. leprae was present in only one of the patients, as all patients had strongly positive skin bacilloscopy. Furthermore, the bacterium was found in a patient with 3 years of disease progression, not in those with 10 or 14 years of disease, as would be expected. As the presence of the bacterium in tissues dependent directly on bacillemia, the assumption is that its presence in the oral cavity may suggest infection of greater severity, but this requires future studies. It was not possible to establish any preferential site for M. leprae in the mouth, as only one patient had specific involvement. Clinical examination of the oral cavity should be routine for leprosy patients, as patterns may be found that may allow the physician to estimate the extension of disease. Although bacilli and granulomas may be present, we emphasize the lack of specific visible lesions in the mouth, while a vast dissemination process occurs in the skin and peripheral nerves. This fact suggests that possibly there may be some factor protecting the oral cavity. Usually visible lesions are seen in long-standing cases, when the disease reaches advanced phases and the body is weakened. Thus, would the oral cavity not be the last line of defense against M. leprae? Would the discovery of this possible protection factor not place us closer to the control of leprosy?

**Table 2:** Histological findings according to site and clinical findings. Dracena, 2002.

Histological findings	Site	Clinical findings	No of patients
Perivascular lymphomononuclear inflammatory infiltrate with no bacilli		Fissures	3
	Tongue	Fissures e edema	1
		Fissures e geographic	1
		Fissures, infiltração e atrofia	1
	Cheek	Macula eritematosa	1
		Macula hipocrômica	1
	Soft palate	Papula	1
		Normal	2

Lymphoplasmocytic inflammatory infiltrate with no bacilli	Soft palate	Normal	1

Granuloma with bacilli	Tongue	Normal	1
	Soft palate	Normal	1

**Figure 1:**
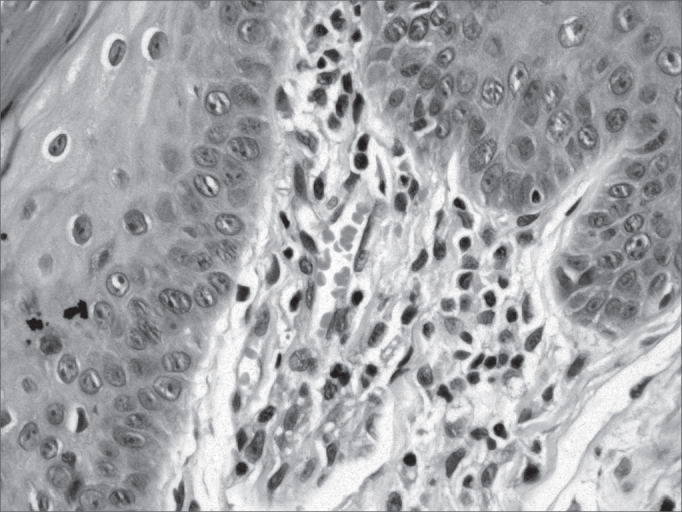
Histopathology of the tongue: Macrophagic sub-epithelial granuloma (HE 400x)

**Figure 2:**
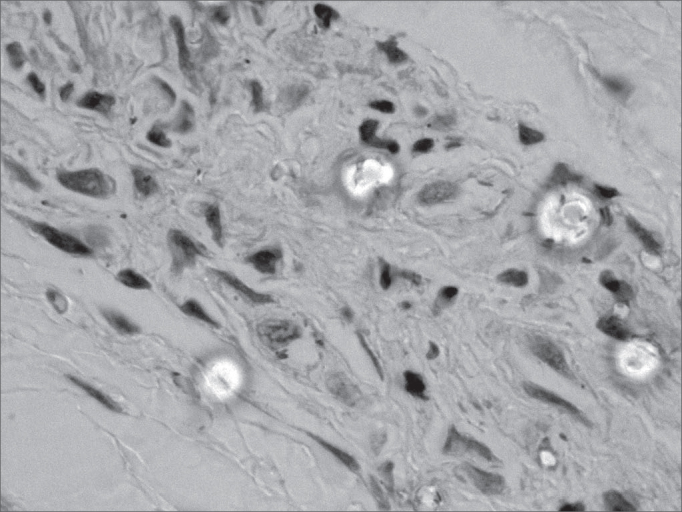
Histopathology of the tongue: Acid-alcohol resistant bacillus (ZN, 1000x)

## CONCLUSION

The clinical and histopathological study of 20 untreated multibacillar leprosy patients with an average disease progression time of 2.5 years allows us the following conclusions:

1. Clinical alteration of the oral cavity does not imply disease involvement; histopathological confirmation is needed.
2. Visible specific lesions of Leprosy are rare in the oral cavity in patients with short duration of the disease.
3. Although signs and symptoms may be absent, the clinically normal mouth in multibacillar cases, even in short term cases, may show specific histopathological involvement.
